# Interrogating the noncoding genome in a high-throughput fashion

**DOI:** 10.1093/nsr/nwy138

**Published:** 2018-11-15

**Authors:** Zhuo Zhou, Wensheng Wei

**Affiliations:** Biomedical Pioneering Innovation Center (BIOPIC), Beijing Advanced Innovation Center for Genomics, Peking-Tsinghua Center for Life Sciences, Peking University Genome Editing Research Center, State Key Laboratory of Protein and Plant Gene Research, School of Life Sciences, Peking University, China

The vast majority of the mammalian genome consists of DNAs that do not encode protein sequences. For decades, the functional potentials of these noncoding DNAs have remained poorly understood. Large-scale studies, such as the Encyclopedia of DNA Elements project and genome-wide association studies, have suggested that the noncoding genome functions in a wide variety of biological and physiological process [1]. However, it has been technically challenging to attribute functions to a plethora of noncoding elements in any given biological context, largely due to a lack of convenient high-throughput approaches.

The recently developed clustered regularly interspaced short palindromic repeats (CRISPR)-Cas system enables efficient and precise perturbation of DNA sequences in the genome, thus offering an unprecedented opportunity to associate functions or phenotypes with genetic elements [2]. Directed by a single-guide RNA (sgRNA) with a region complementary to the target DNA, Cas nuclease cleaves the genomic DNA at the target locus to generate a double-strand DNA break (DSB), which is subsequently repaired through an internal error-prone nonhomologous end-joining (NHEJ) pathway, resulting in an insertion or deletion (indel) that often disrupts gene function [3]. The CRISPR-Cas system has been further engineered to regulate gene expression at will through the fusion of the catalytically inactive Cas9 (dCas9) with transcriptional activators, repressors or other effectors, enabling transcriptional activation (CRISPR activation, CRISPRa), inhibition (CRISPR interference, CRISPRi) or epigenetic modifications [3].

Owing to its programmability and multiplexability, the CRISPR-Cas system is especially potent in high-throughput functional genomics. To achieve this, sgRNAs are designed *in silico* and synthesized as a pool before being cloned into lentiviral vectors to generate a library of viruses for target cell transduction. After phenotypic selection, such as drug resistance/sensitivity or fluorescence-activated cell sorting, candidate genes responsible for the functions of interests are revealed through next-generation sequencing (NGS) analysis of sgRNA barcodes from enriched or depleted cell populations [4].

Despite the power of pooled CRISPR screening in the dissection of key genes in a variety of biological processes, the majority of such screens hitherto have mainly targeted protein-coding genes. This is because the small indels (<10 bp) created by NHEJ are unlikely to produce loss-of-function phenotypes on the noncoding elements. Recently, endeavors have been made to probe the noncoding regions in mammalian genome by exploiting customized CRISPR-based screens.

## FUNCTIONAL SCREENING OF LONG NONCODING RNAS

**Figure 1.**
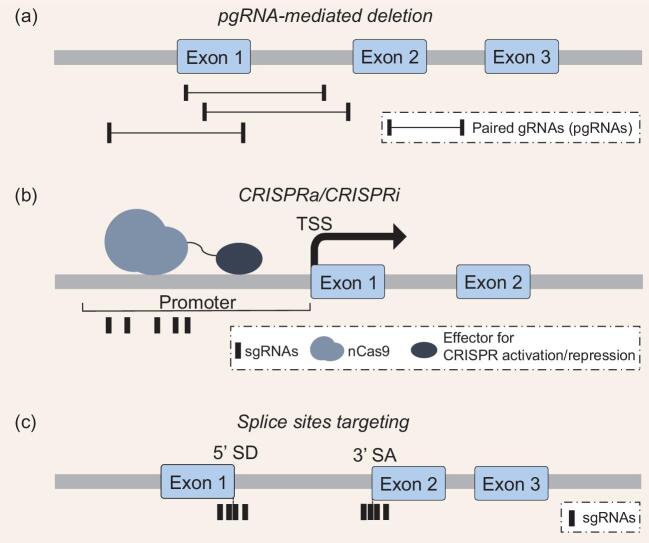
(a) Disruption of lncRNAs by pgRNA-mediated genomic deletion. (b) Perturbation of lncRNA expression by nuclease-dead Cas9 (nCas9)-mediated transcriptional activation (CRISPRa) or repression (CRISPRi). (c) Disruption of lncRNAs by targeting the splice donor (SD) or splice acceptor (SA) sites.

As much as 76% of genomic DNA is transcribed into RNAs, while less than 2% encodes proteins [5]. Long noncoding RNAs (lncRNAs), which are at least 200 nucleotides in length, are the major subsets of the human transcriptome [5]. The first high-throughput method to identify functional lncRNAs is through a specially designed CRISPR approach that employs paired gRNAs (pgRNAs) to produce genomic deletions (Fig. 1a). A pgRNA library comprising 12 472 gRNA pairs specific for 671 human lncRNAs was assembled, and the screening identified 51 lncRNAs that modulate tumor cell growth [6]. Alternatively, CRISPRi and CRISPRa have been employed to investigate functional lncRNAs by perturbing lncRNA transcription in two opposite directions (Fig. 1b). Genome-scale CRISPRi screens were performed in seven different cell lines, using an sgRNA library targeting the transcriptional start site (TSS) of 16 401 lncRNAs. This assay revealed that ∼500 lncRNA loci are important for cell growth [7]. Intriguingly, despite more than 1300 lncRNA genes being expressed in all seven cell lines tested, none of them was identified in all screens, suggesting that lncRNAs exert distinct functions in diverse cellular contexts. Moreover, Joung *et al.* performed a CRISPRa screen to globally map lncRNA loci relevant to drug resistance. By targeting the TSS of more than 10 000 lncRNA loci, 11 were identified whose overexpression conferred cell resistance to BRAF inhibitors [8].

Although lncRNA functional screening at a genome-wide scale could be achieved, the CRISPRi and CRISPRa approaches have limitations, mainly owing to their insufficient perturbation efficiency. Recently, we have devised a new screening approach by specifically targeting splice sites of target genes. Through splicing-targeting to generate either exon skipping or intron retention (Fig. 1c), we established an effective approach to disrupt lncRNA function via an sgRNA. By screening 10 996 lncRNAs in three cell lines, we identified substantial amounts of essential lncRNAs for cellular growth [9]. Alternatively, it is tempting to develop a CRISPR strategy based on base-editing technology [10], since both the 5′ splice donor sites (GT) and the 3′ splice acceptor sites (AG) could potentially be perturbed by base editors, which have been shown to generate A•T>G•C or C•G>T•A conversions in targeted loci [10]. Base editing might be particularly advantageous in negative screens because this approach does not generate DSBs, major sources of nongene-targeting-related cell death.

## MAPPING REGULATORY ELEMENTS

Besides noncoding RNAs, other genomic regulatory elements, such as enhancers, promoters and other unmarked *cis*-acting sequences, play pivotal roles in regulating gene expression [1]. Because Cas nuclease could leave mutagenic footprints, indels within the targeted region, tiling mutagenesis combined with NGS decoding of phenotype-altering sgRNAs has been utilized to identify the approximate sites of sequences important for regulatory elements. This CRISPR-empowered mutagenesis approach has successfully captured key elements in multiple known or putative genomic regions, such as DNase I hypersensitivity sites in the enhancer [11] or confined regions surrounding target genes [12]. However, the tiling library combined with sgRNA sequencing mapped the critical sites at low resolution because the enriched sgRNAs only provided approximate sites of action. Direct sequencing of the mutated region might help reveal sequence-to-function information; however, it is technically challenging to achieve this in a high-throughput fashion with accuracy. A strategy has been reported to repurpose dCas9-activation-induced cytidine deaminase (AID) for protein engineering [13]. While it enables hypermutation *in situ*, the dCas9-AID approach is limited by the presence and density of Cs or Gs on target genes. Recently, by fusing the nCas9 with an error-prone, nick-translating DNA polymerase, Halperin *et al.* achieved highly efficient genomic diversification within a tunable window length in *Escherichia coli*, offering a potential tool for the investigation of the noncoding genome [14].

It is challenging to fine map regulatory elements spanning large genomic regions because of the demand for unrealistic sums of sgRNAs. To map a 2 Mb *POU5F1* locus, Diao *et al.* employed the pgRNA strategy and performed a tiling deletion-based screen. The assay identified 45 regulatory elements, among which 17 were previously annotated [15]. We envisage that the large fragment deletion-based methods have broad applications, such as globally probing the regulatory elements that modulate chromosome architecture, i.e. the active chromatin hub or the CCCTC-binding factor binding sites.

Overall, CRISPR-based high-throughput screening substantially advances our understanding of human noncoding genome architecture and function. With the expansion of the CRISPR toolbox, such as the Cas9 variants with broadened protospacer adjacent motif compatibility and higher specificity, if proven effective [16], a more precise and detailed functional map for the noncoding genome could be delineated in the near future.
